# Remote teaching during the COVID-19 pandemic: repercussions from professors’ perspective

**DOI:** 10.1590/0034-7167-2022-0172

**Published:** 2023-02-03

**Authors:** Joanara Rozane da Fontoura Winters, Débora Rinaldi Nogueira, Ivonete Terezinha Schülter Buss Heidemann, Michelle Kuntz Durand, Adriana Bitencourt Magagnin, Aline Megumi Arakawa-Belaunde

**Affiliations:** I Instituto Federal de Santa Catarina. Joinville, Santa Catarina, Brazil; II Universidade Federal de Santa Catarina. Florianópolis, Santa Catarina, Brazil

**Keywords:** Social Isolation, COVID-19, Mental Health, Faculty, Teaching., Aislamiento Social, COVID-19, Salud Mental, Docentes, Enseñanza., Isolamento Social, Pandemias por COVID-19, Saúde Mental, Docentes, Ensino.

## Abstract

**Objectives:**

to understand the repercussions of teaching work in remote teaching during the COVID-19 pandemic in Higher Education Institutions in northern Santa Catarina.

**Methods:**

a qualitative participatory action research, based on Paulo Freire’s theoretical-methodological precepts. Seventeen health professors participated in two Virtual Culture Circles held in the first half of 2021.

**Results:**

six generating themes emerged for discussion, which aroused in participants’ feelings and perspectives regarding the remote teaching process in the pandemic context, with an emphasis on the connectivity theme, which generated dialogue through reports of personal experiences.

**Final Considerations:**

the pandemic has had repercussions in sectors such as health and education. Professors talked about their experience in creating, recreating and adapting to remote teaching and the challenges facing the teaching-learning process, listing the worsening of mental health and the need to learn new digital technologies.

## RESUMO

**Objetivos:**

compreender as repercussões do trabalho docente no ensino remoto durante a pandemia de COVID-19 em Instituições Superiores de Ensino do Norte de Santa Catarina.

**Métodos:**

estudo qualitativo, tipo ação-participante, fundamentado nos preceitos teórico-metodológicos de Paulo Freire. Participaram 17 docentes da área da saúde em dois Círculos de Cultura Virtual realizados no primeiro semestre de 2021.

**Resultados:**

emergiram seis temas geradores para discussão, os quais despertaram nos participantes sentimentos e perspectivas em relação ao processo do ensino remoto no contexto pandêmico, com ênfase à temática conectividade, que gerou o diálogo por meio de relatos de experiências pessoais.

**Considerações Finais:**

a pandemia trouxe repercussões em setores como a saúde e a educação. Os docentes dialogaram sobre sua vivência em criar, recriar e se adaptar ao ensino remoto e os desafios frente ao processo ensino-aprendizagem, elencando o agravamento da saúde mental e a necessidade de aprender novas tecnologias digitais.

## RESUMEN

**Objetivos:**

comprender las repercusiones del trabajo docente en la enseñanza a distancia durante la pandemia de COVID-19 en las Instituciones de Educación Superior del Norte de Santa Catarina.

**Métodos:**

estudio cualitativo, tipo acción-participante, basado en los preceptos teórico-metodológicos de Paulo Freire. 17 profesores de salud participaron en dos Círculos Virtuales de Cultura realizados en el primer semestre de 2021.

**Resultados:**

surgieron seis temas generadores de discusión, que despertaron en los participantes sentimientos y perspectivas respecto al proceso de enseñanza a distancia en el contexto de la pandemia, con énfasis en el tema conectividad, que generó diálogo a través de relatos de experiencias personales.

**Consideraciones Finales:**

la pandemia ha tenido repercusiones en sectores como la salud y la educación. Los docentes hablaron sobre su experiencia en la creación, recreación y adaptación a la enseñanza a distancia y los desafíos que enfrenta el proceso de enseñanza-aprendizaje, enumerando el empeoramiento de la salud mental y la necesidad de aprender nuevas tecnologías digitales.

## INTRODUCTION

On December 31, 2019, the World Health Organization (WHO) was alerted to a virus causing pneumonia in Wuhan City, Hubei Province, People’s Republic of China. It was a new strain (type) of coronavirus that had not been identified before in humans^(1)^. On January 30, 2020, WHO reported that Coronavirus Disease 2019 (COVID-19) was a public health emergency^(1)^.

In this regard, there were several precautions to prevent the virus from spreading, including social distancing, the use of masks and hand washing. In Brazil, the first cases were confirmed in February and several actions were also taken to prevent the disease from spreading. However, a Public Health Concern of National Importance (PHCNI) was declared^(2)^.

The COVID-19 pandemic has brought changes in world daily life due to health measures and social isolation, which is essential. Therefore, all sectors were affected: industry, commerce, agriculture, service sector; among the most committed sectors, education stands out. Professors had the task of educating, considering the inequalities of access to information technologies by students, so professors and students had to adapt to a new form of teaching: remote teaching^(3)^.

Thus, emergency remote teaching was a possibility that educational institutions used to repair a crisis situation, however it should not be compared with Distance Learning (DL). DL is the educational modality in which the didactic-pedagogical mediation in the teaching and learning processes occurs with the use of information and communication means and technologies, with students and professors developing educational activities in different places or times^(4-6)^. Professors came across remote classes, which proved to be less productive over time, compared to face-to-face classes, requiring active teaching and learning strategies, which requires significant changes in teaching practice^(7)^.

These changes in professors’ education and way of teaching the class were a paradigm break, as professors had to quickly adapt and teach content from their face-to-face classes to online platforms with the use of Information and Communication Technologies (ICT)^(8-9)^.

It was found that, wishing or not, professors found themselves included in the context of a crisis, impelling them to invest in the challenge of developing skills of interaction, education, communication and use of technologies^(7)^.

With teaching in the remote format, the teaching-learning process has changed, weakening the collectives of workers that, historically, have strengthened the workplace itself, as a place for meeting and carrying out individual and collective activities^(10)^.

In this new teaching modality, professors found themselves living a ‘solitary teaching’ in the current scenario, causing changes in teaching practice and some degree of psychological distress.

The pedagogical space shared between professors and students in which socialization, movement and dialogue occurred began to be replaced by a virtual, restricted and solitary encounter, which could generate dissatisfaction, sadness and anxiety^(10-11)^.

It is necessary to emphasize that nursing and other health courses lack interaction, exchange of practical knowledge that is inherent to training; however, the remote emergency education adopted, in the course of social isolation, caused by the COVID-19 pandemic, required adjustments to the teaching process, which may point us to new circumstances of physical and mental exhaustion of professors and students^(12)^.

## OBJECTIVES

To understand the repercussions of teaching work in remote teaching during the COVID-19 pandemic in Higher Education Institutions in northern Santa Catarina.

## METHODS

### Ethical aspects

The ethical aspects of this research, following CNS Resolution 466/2012, were respected, with data collection starting only after approval by the Research Ethics Committee. The project was approved in 2020. To preserve anonymity, professors were named according to the teachings of Paulo Freire, and as a reference, the book “*Pedagogia da Autonomia*”^(13)^ was used.

### Theoretical-methodological framework

It was based on Paulo Freire’s theoretical and methodological framework. Paulo Freire’s Research Itinerary was used, which comprises three interdependent stages: 1) thematic investigation; 2) coding and decoding; 3) critical unveiling^(14)^.

### Methodological procedures

**Figure 1:**
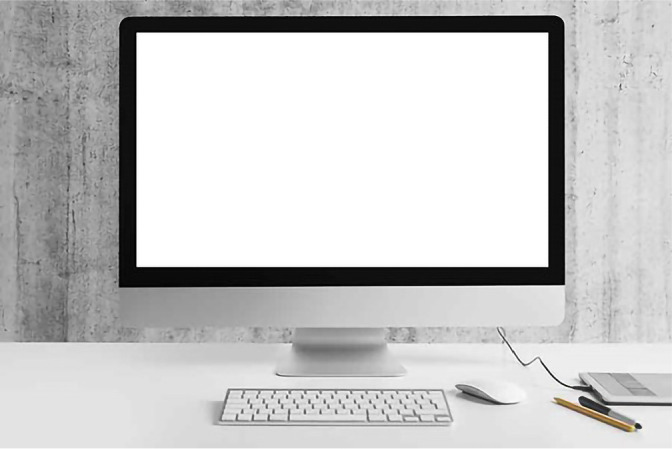
Paulo Freire’s Research Itinerary: analogy with the computer

*Source: image adapted by the authors from https://gartic.com.br/amanda_sccp/desenho-jogo/lapis-de-cor.*

To go through the stages of Paulo Freire’s Research Itinerary in a concrete, interactive way, critical, creative and playful, an analogy was made with the computer, as shown in Figure 1.

**Figure 2:**
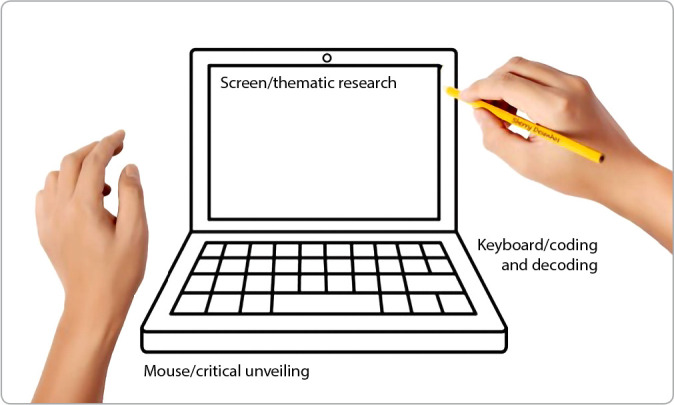
Search Itinerary Representation

*Source: extracted from the Virtual Culture Circle with professors. Image adapted by the authors from an image of the internet.*

To start the Virtual Culture Circle (VCC) and go through the first phase of Paulo Freire’s Research Itinerary, which is the thematic investigation, the mediators shared the image of a computer that would symbolize remote teaching (Figure 2). They explained that the screen would represent the thematic investigation, which would be the universe of professors; the keyboard, with the letter options, would symbolize the meanings that would emerge from the ideas, from the thoughts, allowing the possibility of coding and decoding the themes, thus requiring a better understanding; the mouse would portray the critical unveiling, in which the subjectivity of professors’ thoughts is revealed.

Based on the representation, professors were encouraged to express significant themes related to the COVID-19 pandemic, raising the following questions: what is it like to live teaching work in remote teaching? What are the powers, facilities and positive events of this remote moment? What are the weaknesses, difficulties and negative events of this remote moment? Professors talked about the questions, and their perceptions, experiences and knowledge were digitized on the computer screen. As participants raised the themes, a mediator wrote down the answers regarding the questions, encouraging them to organize them according to the subjects addressed by the group.

### Study design

This is qualitative participatory action research^(15)^, in which Paulo Freire’s Research Itinerary was used, which takes place in a Culture Circle space, which enables the exchange of experiences, linked to the construction of knowledge. Participants dialogue on common themes in a horizontal and participatory way, stimulating collective knowledge in search of the transformation of reality^(16)^.

### Study setting

The study was conducted at three Higher Education Institutions in the state of Santa Catarina. Seventeen professors participated in the research, including professors of courses in the health area who were working in remote education in the COVID-19 pandemic. Professors who were on leave during the study period were excluded.

Due to the pandemic context, it was decided to perform the VCC, configuring a practice of great challenges and innovative. Thus, the institutional Google Meet application was used.

### Data collection and organization

The strategy to obtain the data was the VCC. Professors from educational institutions were invited via e-mail. After accepting to participate in the study, a message was sent via WhatsApp, scheduling the VCC according to participants’ availability. Then, the Informed Consent Form (ICF) was sent via e-mail, which they signed and forwarded to the researchers.

Two VCCs were performed through Google Meet, lasting approximately two hours and an interval of one month between them. The meetings took place in the first half of 2021.

The tools used during the Research Itinerary stages were compared to the image of a computer. After completing the VCC, the dialogues were transcribed and organized according to the methodological framework.

### Data analysis

To validate these records, all the sentences were re-read for professors, motivating them to continue their reflections on the proposed themes, with a view to sealing the action-reflection-action process, encouraging them to understand their ability to face the challenges raised and share proposals that make it possible to transform their reality^(14)^.

For critical unveiling, the mediators approached that the computer represents remote teaching, and professors were invited to reflect on everything they said and heard from others, in order to unveil the real possibilities to live with health and overcome the challenges of COVID-19 in the teaching and learning process, seeking to transform their difficulties and facilities at this time. The process of unveiling themes occurred concurrently with the thematic investigation, as foreseen in Paulo Freire’s Research Itinerary assumptions, which foresees the analytical process^(14)^.

## RESULTS

The VCC had the participation of 17 people linked to Higher Education Institutions, 14 females and three males, with an average age 30 to 60 years. Participant training occurred predominantly in the area of nursing, residing in different municipalities in the state of Santa Catarina, Brazil.

The first phase culminated in the identification of six generating themes, forwarded to the encoding and decoding stage, which were: connectivity; concern for the future; solitude/solitary teaching; apprenticeship; impaired dialogue; and organizational difficulty. These themes reflect participants’ reality of life, through their speeches and emotions expressed. In order to organize and facilitate the accomplishment of Paulo Freire’s Research Itinerary stages, the generating themes and encodings were reproduced on slides during the virtual meeting.

Participants revealed their feelings and perspectives in relation to the remote teaching process at the pandemic moment, reflected throughout the dialogues carried out in reports of personal experiences. Among the generating themes, connectivity was a predominant theme, resulting in several concerns and questions among the participants, raising moments of reflection. Thus, in this study, connectivity was highlighted, relating the positive aspects of remote teaching, the use of technologies and their limits observed by participants.

Professors discussed how the use of online tools would provide relationships of approximation that, previously, did not occur or that distance ended up making it difficult:

[…] *just as our emotions fluctuate, our relationship with remote teaching fluctuates. At first, everything seemed very interesting, innovative, and possible to broaden horizons. We could use new tools, bring people from far away, so it all seemed very interesting.* (Lovingness)

[…] *but I also feel, in contrast, that I learned a lot, so I feel like a much more connected person today. So, I learned to take classes in different programs that I didn’t even know existed. So, in addition to all these challenges, I feel that it was a great learning moment for me to use other tools, to teach in a different way.* (Freedom)

Remote teaching required from professors a drastic change in the way of teaching, because they had to master and learn new pedagogical practices, using various technological devices. Students had to adapt to the new digital reality, although this technology was already inserted in their daily lives; however, they had to stay in front of the computer for a longer period, to participate in the classes. In the dialogue in the Culture Circle, the participants demonstrated the need to seek new adaptations of the classes, to maintain students’ involvement and interest. This also applied to the relationship between the professors themselves and the institution, in order to maintain the necessary alignments for the work processes, as reported in the statements below:

[…] *I also thought that, in a way, Distance Education has given opportunities* […] *it has taught* […] *different views of teaching,* […] *and I was very used to looking at teaching as something professor-student. And it helped to have a little more independence, to go further, to search for the content alone.* (Criticality)

[…] *we have developed mechanisms and resources to improve our communication with students, with professors, with the institution, so I understand that it is quite positive.* (Esthetic)

They also highlighted training opportunities and discussions that occur virtually, enabling the participation of all and continuous updating.

[…] *I also remembered the opportunity we had to participate in online events, listen to professionals and professors that we would not be able to without going to a conference very far away, very expensive, and we had this access* […]. (Generosity)

Participants stated that remote teaching provided approximation, interaction, savings and time management, as reported:

[…] *meeting different people and interacting with people from different universities* […]. (Hope)

However, when working from home, the participants highlighted as positive aspects improvement in diet, adequate sleep habits, preparation of their own meals, inclusion of healthy habits in the routine, infusions and teas, physical activity and proximity to the family.

As significant themes involving connectivity, highlights access to communication, which could be a facilitator, however this theme also included negative aspects, and may be considered limits in the routines of professors. This unfavorable aspect was due to the interlocutors’ difficulty in understanding limits related to time and space in accessing the other, the immediacy in the answers, in addition to the concomitant activities carried out without pauses and rests.

[…] *the other thing that I see as a point of negative connectivity, which I think weighs a lot, including in my organizational issue, is the ease that people have to communicate with you, regardless of the time, regardless of the day, of the functions. Access, requiring an immediate response to some situations, became much easier and, here, the anxiety of a more immediate response is more fortified, I think so.* (Consciousness)

[…] *this issue of people not even having the sense to send you midnight, Saturday, Sunday, at lunchtime. So, as if we had an obligation to be “on” twenty-four hours, regardless of the time.* (Hope)

[…] *So, I think that’s it: we don’t disconnect, both in the sense of communicating with each other, of sending messages as well as receiving these messages. So, we have to police ourselves in both directions, in this back-and-forth relationship, both not sending and also trying not to read.* (Autonomy)

The possibilities of performing many actions or even simultaneous led people to exhaustion conditions, generating feelings of demotivation when developing/participating in remote actions.

[…] *connectivity is so wide and makes accessing things so easy that we access several things at the same time, we start doing one thing after another, one activity after another, without a break* […]. (Consciousness)

[…] *meetings are from noon to 1:45 pm. Lunch time for professionals. The insanity we’re coming.* (Autonomy)

[…] *various online events: lives, webinars, lectures. So much cool stuff going on, but I can’t seem to do anything.* (Consciousness)

In addition, the teaching commitment was present in the possibility brought by connectivity related to the aspect of tiredness and the need for immediate return.

[…] *weekend, and students sending questions, you know? Sunday. So, I thought “no, I won’t even look”, because if I open it, I’ll answer. I also can’t stand to see the student’s question and not answer.* (Autonomy)

The physical impact of remote actions was scored due to pain, such as in the ear and difficulty of acoustic feedback. This condition indirectly influenced professors’ vocal production, who began to demand more from their voice, which could generate discomfort and impact on vocal quality.

[…] *and another thing too: this phone issue. Ear pain I already have because of the abusive use of headphones, I notice it, it’s already very painful, and we talk louder. Now I’m trying to do it here: put it on one side, to see if I can speak more quietly, because sometimes my daughter says “mom, you’re talking too loud”, and then I realize that it’s because I’m with headset. And then, in the dialogue inside the house, the tone of voice remains high.* (Autonomy)

When reflecting on the impacts of connectivity, participants highlighted the overload, such as the permission to carry out the activities:

[…] *allowing ourselves and allowing ourselves to allow ourselves, to make connectivity go beyond our personal situation* […], […] *at the time of working the no’s. Do I know how to say no? Can I say no? This is the time for us to say no.* (Humility)

Connectivity has been present in the daily lives of many people, but it can also lead to an “overload” of information, which generates anxiety and stress, as observed in previous statements. One participant reported that, at times, she sought to move away from the devices that kept her connected, such as TV, computer and smartphone, thus allowing moments of pause from her activities.

Through theme unveiling regarding connectivity, participants referred to possible actions to be directed, particularly to negative aspects. The action-reflection-action process allowed the group to understand the need to affirm some positions. They unveiled their own boundaries, of disconnecting, shutting down the internet.

[…] *connectivity is doing in our life. And some unveiling strategies that we can be working on to improve our life, and we were connected to it, but we weren’t looking.* (Curiosity)

The positive points of connectivity were resumed as a way of seeking to overcome the difficulties experienced in the daily practice of professors amidst the pandemic situation experienced.

[…] *I perceive it* [connectivity] *as something very positive, right, in terms of geographical barriers, of having access* […] *the ability for everyone to be able to connect at the same time and watch at the moment, participate in the same conversation.* (Curiosity)

During the group’s reflection, about thoughts that could reveal the theme, the discussion was also raised about the necessary exercise so that people do not have their thoughts fixed on the uncertainties of the future, thus bringing their lives to the present and seeking the best way to adapt to the difficulties experienced.

As one of the negative aspects of connectivity was the increased perception of anxiety and stress, some statements suggested the removal of social networks excessively:

[…] *so, I try to avoid some types of news and some sites that I used to access, and I avoid going. And that tip, right, for us to try to stay and live more in the present, so let’s live in the now. What is the context of now? Now we are remote and, in the future, we will see how things will happen and how we will adapt to the new context that will emerge.* (Freedom)

The group considered the negative implications for students’ learning process; however, it reaffirmed that the pandemic has brought challenges to all people, but that it may still be possible to seek training based on students’ needs:

[…] *try not to think that everything is fine, that teaching is going smoothly, that there is no weakness, but to look at it and think about how it can be improved. How is this student receiving, I don’t know if it’s that word, but just as he is acting in this process, how he is seeing himself in this process, how he is receiving this content*. (Curiosity)

At the end of discussion, some necessary actions for individual and collective changes were pointed out so that professors could deal with the challenges of work during the pandemic. The group raised the importance of sleep hygiene, the reduction of access to applications and networks that are involved with work in their moments of rest, the reduction of time in front of television or on websites with bad news, the enhancement of dialogue between peers for the exchange of experiences and mutual help in the teaching-learning process.

## DISCUSSION

According to the WHO statement regarding the first confirmed victim of COVID-19 in Brazil, a series of repercussions and intersectoral concerns began in the country. Pervading the health sector, several scenarios were affected and required a reorganization to adapt to the pandemic demands^(17)^.

On March 17, 2020, through Ordinance 343, the Ministry of Education replaced face-to-face classes with digital classes during the COVID-19^(18)^ pandemic situation. This measure was taken as a result of the first cases of COVID-19 in Brazil; thus, a new proposal for education and work was being conceived and everyone had to adapt to the new model of organization of society: people, including professors, working from home office^(19)^.

Faced with this new proposal, educational institutions had to reformulate their Course Pedagogical Projects (CPP), teaching plans and adopt the remote class model subsidized by technological and digital resources. Thus, this new education was a temporary solution to a particular emergency situation^(20)^.

Remote education, the possibility for students to be in a different geographical space from professors, was implemented in an emergency, because professors and students were prohibited by municipal, state and federal decrees from attending educational institutions, to prevent the virus from spreading^(21)^.

Professors were used to face-to-face classes and, most of the time, they did not need to use many technological or digital resources, they had to leave their face-to-face universe, in which they had mastery, and reinvent themselves, because the vast majority were not prepared or qualified for it^(22-23)^. In a study carried out with professors on non-face-to-face teaching, it was highlighted that this generated insecurity and fear, lacking greater training for the use of virtual technologies^(24)^.

Therefore, there were great changes, both for professors and students, because the way of teaching and learning was drastically modified, observing a rupture in the pedagogical pattern^(20)^.

However, this is a moment of reflection and change, and every change requires patience, study, motivation, break of paradigms and knowledge of the new; it requires curiosity, critical consciousness, and technology brings stimuli and challenges to curiosity. Thus, professors learn, teach and awaken critical reflection and dialogue in students. Therefore, to paraphrase Paulo Freire^(13)^, a good professor is what manages, while speaking, to bring students to the intimacy of the movement of their thought. Regardless of the mode, whether face-to-face or remotely, professors manage to awaken critical consciousness, curiosity and the search for knowledge in students^(19)^.

Thus, the different challenges to put into practice other modes of teaching required professors to plan, synchronous and asynchronous classes, and the elaboration of pedagogical strategies, allowing there to be both emotional exhaustion and discouragement and other situations of aggravation to their health^(22)^.

Corroborating other studies that assessed the impact of the pandemic, this research demonstrates implications for professors’ mental health. Adaptation to a new way of life culminated in interference with people’s mental health, including changes in sleep quality, anxiety and other depressive symptoms.

Some factors may potentiate this feeling of overload, such as the previous history of anxiety, exaggerated work pace, professional devaluation, scrapping of the sector and working conditions that do not correspond to service demands. Since the exercise of the profession represents a large part of people’s lives, it is necessary to look at the elements that are negatively influencing the quality of life of professors^(25)^.

The balance between gains and losses amidst the pandemic context impacted professors’ mental health, in the face of the sudden migration of remote class and the immediate adaptation of the forms of teaching, content and skills, such as in the deepening of the use of information and communication technologies, aspects that in themselves generate work overload related to the concern with the quality of teaching^(26)^.

The cognitive demand that the teaching work requires, associated with a poor quality of sleep, can have a direct impact on professional performance and satisfaction, affecting greater psychological distress. This, in turn, can be accentuated in the face of gender differences, since, in the sexual division of work, the multiple tasks are absorbed by the woman, which increases the working day, considering household chores and child care^(27)^.

It is noticed that the pandemic context imposed on professors a deep reorganization of their work routines. The physical barrier between work and family life, at home, ceased to exist and, in most cases, forced various improvisations to the family and domestic routine, to allow the minimum structure necessary for teaching-learning activities. The remodeling of the ways of exercising teaching and the acute revision of working time and family life produced significant negative consequences on professors’ physical and mental health. The scarce daily time dedicated to rest and high proportions of poor sleep quality were some of the health situations identified in the context of the pandemic^(27-28)^.

Remote work enabled that positive aspects are also presented, such as enhancing the professional development of professors by acquiring new skills in the professor-student dyad, optimizing time in the face of decreasing distances, approaching the family context and breaking geographical barriers^(10,25)^. It is essential, therefore, to offer continued support to professors to bring the relationship with the new information and communication technologies closer, thus avoiding the feeling of powerlessness in the face of the challenges related to this teaching modality^(29)^.

Moreover, the weakening, devaluation, suffering and illness of the teaching work was noticed, in which the search for their autonomy is related to forms of care centered on professors. The relationships between autonomy and care permeate aspects of teaching workers’ health, which can contribute to interdisciplinarity in their health care and key to the discussion of a culture of care both in virtual and face-to-face work^(30)^.

### Study limitations

The study had as a limiting factor the low participant compliance in carrying out the VCC. It is believed that, due to professors’ excessive unexpected work, the challenge is to reconcile a schedule with all professors to carry out the VCC, due to accumulation of activities that they develop in their work process.

### Contributions to health

This study contributes to knowing the opinions of professors related to remote teaching and its repercussions on these professionals’ mental health during the pandemic, as well as directing discussions on the subject in educational institutions, sharing successful experiences in remote teaching during the pandemic.

## FINAL CONSIDERATIONS

During the VCC, professors talked about their experience in creating, recreating and adapting to remote teaching. They discussed constant connectivity and access to communication as situations that need to work to make online education feasible during the COVID-19 pandemic.

They highlighted challenges facing the teaching-learning process, listing the worsening of mental health and the need to learn new digital technologies. They highlighted insecurity and fear in learning to use technological tools and how this modality of virtual teaching interfered in the daily life of each professor, such as invasion of private space, quality of sleep and lack of time limits dedicated to work and leisure. They highlighted the use of online tools, promoting relationships of approximation between people far away geographically.

The dialogue in the Culture Circles made it possible to reflect how the COVID-19 pandemic is interfering with professors’ health. Through Paulo Freire’s Research Itinerary, an understanding of the repercussions of worse mental health and the necessary care was revealed. Through the Culture Circles, it was possible to share congruent experiences and motivations among peers, care in a collective way, reinforcing this space of mediation and resilience.
